# The impact of assistive devices on community-dwelling older adults and their informal caregivers: a systematic review

**DOI:** 10.1186/s12877-022-03557-8

**Published:** 2022-11-24

**Authors:** Keshini Madara Marasinghe, Ashok Chaurasia, Maisha Adil, Qian Yue Liu, Teeyaa Ibrahim Nur, Mark Oremus

**Affiliations:** 1 School of Public Health Sciences, University of Waterloo, 200 University Avenue West, Waterloo, ON Canada; 2 Oxford Institute of Population Ageing, University of Oxford, Oxford, UK; 3 UBC Hospital, 2211 Wesbrook Mall, Vancouver, BC Canada

**Keywords:** Assistive devices, Assistive technologies, Community-dwelling older adults, Informal caregivers, Systematic review

## Abstract

**Objective:**

The purpose of this systematic review is to assess the impact of assistive devices on the life satisfaction of (Research Question 1), and informal caregiving hours received by (Research Question 2), community-dwelling older adults (≥ 65 years).

**Methods:**

We searched CINAHL, MEDLINE, and Scopus from database inception to March 2022. For each question, two reviewers independently screened citations, extracted and narratively synthesized the data, and assessed article quality and strength of evidence.

**Results:**

Of the 1391 citations screened, we found two articles pertaining to each question, for a total of four articles. In general, assistive device use was not associated with life satisfaction, while it was positively associated with informal caregiving hours. However, the risk of bias was serious across the two studies for Research Question 1, and the overall quality of evidence was “very low”. The risk of bias was not serious across the two studies included in Research Question 2 and the overall quality of evidence was “low”.

**Conclusion:**

Due to the scarcity of studies, the limitations of existing studies (i.e., risk of bias), and the evidence being low or very low quality, we could not draw firm conclusions about the associations of interest. Additional research will produce a better understanding of the two relationships and provide further evidence to inform policy decisions regarding the provision and funding of assistive devices for community-dwelling older adults.

**Trial registration:**

This systematic review was registered in the International Prospective Register of Systematic Reviews (PROSPERO) database of systematic reviews (identification number: CRD42021248929).

**Supplementary Information:**

The online version contains supplementary material available at 10.1186/s12877-022-03557-8.

## Background

### Assistive devices

The World Health Organization (WHO) defines assistive devices (AD) as “devices and technologies whose primary purpose is to maintain or improve an individual’s functioning and independence, to facilitate participation, and to enhance overall well-being” [1]. Examples of AD include, but are not limited to, mobility devices such as wheelchairs, walkers, canes; visual devices such as magnifiers, white canes, Braille reading materials; audio enhancement devices such as hearing aids and amplifiers, and communication and information management devices such as software and apps [2].

Previous studies have found that AD contribute to the overall well-being of community-dwelling older adults by preventing impairment, delaying hospitalization, slowing the decline of functional and cognitive abilities, and improving independence and ease of living, social connectivity, safety, and mental health. However, not all features of AD have been extensively investigated across different populations and settings [1–10]. Two such features are the potential of AD in improving life satisfaction of, and reducing informal caregiving hours received by, community-dwelling older adults.

#### The aging population

The world population is aging. In 2050, one in every six persons in the world will be above the age of 65 years, with the absolute number of older persons reaching approximately 1.5 billion [11, 12]. One reason for the increasing global population of older adults is simply that people are living longer. By 2040, global life expectancy is expected to rise by 4.4 years [13]. In the years 2015–2020, a person aged 65 years could live an additional 17 years on average, which is predicted to rise to 19 years by 2045–2050, globally [11]. However, while people are living longer, not all years are lived in full health. The average number of years of healthy life lost to poor health has risen from 8.62 in 1995 to 9.72 in 2017, and is expected to increase in the majority of countries [14].

### Life satisfaction (LS)

Life satisfaction (or ‘satisfaction of life, ‘satisfaction with life’) is defined as “cognitively oriented, subjective judgment of one’s life as a whole and current life situation in relation to one’s own expectations” [15–18]. LS is a key indicator of a person’s normative opinion about their overall well-being [19, 20]. The concepts of LS and quality of life are sometimes used interchangeably in the literature. Although related, they are separate, with quality of life pertaining to a holistic conception of one’s state of life that encompasses physical and psychological health, degree of independent functioning, social engagement, personal views on health, and the relationship between individuals, their health, and the environment [21, 22]. Diener’s classical theory of LS suggests that LS is expected to decline with age, in tandem with other elements of life such as health, finances, work, and family [23]. According to this theory, reductions in LS are likely to occur as adverse health conditions become more prevalent among populations that are rapidly aging and living longer.

While it may not be immediately possible to eliminate or improve health challenges that appear in later life, one can take steps to minimize the impact of these challenges through adaptation, e.g., using AD to reduce the impact of mobility impairment. Such steps can improve the state and experience of living as an older adult, thereby enhancing LS. Previous research has also found that greater LS is associated with positive health outcomes (i.e., better physical/psychosocial health, and health behaviours), whereas lower LS is linked with negative outcomes (i.e., high incidence of chronic conditions, hospitalization, and mortality) [24, 25].

#### Assistive devices and life satisfaction

The link between AD and LS is recognized in the Consortium for Assistive Technology Outcomes Research (CATOR) framework [26]. CATOR identifies subjective well-being as an outcome. Within this framework, LS refers to how persons who utilize AD, value AD and believe these devices influence their LS [26]. According to this framework, AD may have the potential to improve LS in older adults.

#### Informal caregiving hours

Our second research question examines the association between AD use among community-dwelling older adults and informal caregiving hours received by these adults. Informal care refers to unpaid care and assistance provided by family, friends, or neighbours to those who require assistance due to physical, cognitive or mental conditions [27]. Amid populations that are aging and living longer with comorbidities, requirements for such assistance are growing and the number of older adults who will require informal care in Canada is expected to increase by 1.2 times between now and 2050 [28].

Caregiving hours is an important marker of the intensity of informal care and is a risk factor for caregiving stress/burden [29, 30]. While other factors besides caregiving hours contribute to caregiver stress/burden (e.g., care recipient’s dependency level), these factors were beyond the scope of the review [31]. Research has shown that stress related to informal caregiving is positively associated with the number of care hours provided [32–35]. Higher numbers of caregiving hours may also pose physical, emotional, financial, and social challenges for informal caregivers, leading to an accelerated deterioration of their overall health and well-being [36]. Studies of informal caregivers from countries like Canada, the United States, Australia, and the United Kingdom have reported positive associations between high informal caregiving hours and lack of exercise, unhealthy eating, alcohol consumption, mobility limitations, caregiver stress, depression, anxiety, long-term back problems, pain or discomfort, low quality of life, lack of personal or family time, and overall poor health [36–41].

### Assistive devices and informal caregiving hours

According to CATOR, social significance refers to the impact of AD on society and other people (i.e., caregivers) [26], including the nature and amount of effort put into caring for persons who utilize AD [26, 42]. The CATOR framework lays the foundation for investigating the relationship between AD use and informal caregiving hours.

We undertook this systematic review to examine the existing literature on AD use and: 1) the LS of community-dwelling older adults (≥ 65 years of age) who utilize AD (Research Question 1); and 2) informal caregiving hours received by community-dwelling older adults (≥ 65 years of age) who use AD (Research Question 2).

## Methods

### Protocol registration

This systematic review was conducted and reported in accordance with the Preferred Reporting Items for Systematic reviews and Meta-Analyses (PRISMA) guidelines (See Additional file 1: APPENDIX A) and registered in the International Prospective Register of Systematic Reviews (PROSPERO) database of systematic reviews (identification number: CRD42021248929) [43].

### Database search and search strategy

The database search and search strategy were developed in consultation with a health sciences librarian. Keywords related to four concepts (AD, LS, informal caregiving hours, and older adults) were used for the database search, which covered CINAHL (1961 to March 2022), MEDLINE (1950 to March 2022), and Scopus (1966 to March 2022) (See Additional file 1: APPENDIX B). The search strategy syntax was developed for Scopus and adapted to the other databases.

After removing a total of 47 and 38 duplicates for Research Question 1 and Research Question 2 respectively, two reviewers independently screened the titles and abstracts of the remaining citations, as well as the full texts of papers that passed title and abstract screening. The reviewers resolved discrepancies by consensus. The references of included articles were examined to identify other relevant articles, which were put through the screening process.

#### Inclusion criteria

Reviewers searched for peer-reviewed, quantitative articles that included a parallel comparison group and used the following eligibility criteria to screen these articles for relevance to the research questions:

Objectives 1 and 2 included articles that focused on: any AD that falls under the International Organization for Standardization (ISO) defined 12 classes of AD, including a wide range of devices such as mobility and sensory aids, mHealth devices (e.g., software applications to enhance memory), among others (See Additional file 1: APPENDIX C); community-dwelling older adults who utilize AD and are 65 years of age or older; and articles written in any language. Research Question 1 considered articles that investigated the impact of AD on the LS of persons who utilize devices, whereas Research Question 2 included articles that explored the association between AD use and informal caregiving hours among carers aged 18 years or older.

#### Exclusion criteria

We excluded commentaries, letters to the editor, pre-post studies, case series, abstracts, and animal studies.

### Data extraction, analysis, and quality assessment

At least two reviewers independently extracted the following information from each study: general (i.e., year, country, follow-up duration, authors, intervention), characteristics of the sample (i.e., mean age, sample size, sex, setting, population), and outcome measures (i.e., LS scores and the number of informal care hours (See Additional file 1: APPENDIX D).

A meta-analysis was not conducted due to the heterogeneity of included studies. Heterogeneity arose from variations in tools used to measure exposure and different methods of reporting quantitative results, e.g., *p*-values, test statistics, regression coefficients. The components necessary to convert the results into a common metric were not reported in all the included studies. Instead of a meta-analysis, we narratively synthesized the data. We used the ‘esc’ package in R v 4.2.0 (The R Foundation for Statistical Computing, Vienna, Austria) to calculate Hedges’ g in one study. Results from all included articles were covered in the narrative synthesis.

Risk of bias was assessed with the Appraisal Tool for Cross-Sectional Studies (AXIS) [44]. All AXIS questions can be answered with “yes”, “no”, or “don’t know” and we awarded 1 point for each ‘yes’ response. In addition to the traditional scoring of AXIS, we chose to award 0.5 points for a ‘partial yes’. Question 14 (describing non-responders) was not scored and instead it was classed as a sub-question of question 13 (concerns about non-response bias), resulting in a maximum score of 19 points (See Additional file 1: APPENDIX E) [45, 46].

The quality and strength of evidence of the included articles was rated using the Grading of Recommendation, Assessment, Development, and Evaluation (GRADE) approach [47]. GRADE creates evidence summaries and builds refined recommendations transparently and systematically [47]. The certainty of evidence is evaluated based on factors such as risk of bias, indirectness, inconsistency, imprecision, publication bias, and confounding [47].

Upon completion of the review, we self-rated the quality of the review using the assessment of multiple systematic reviews (AMSTAR) 2 [48], omitting the randomized controlled trial portion of question 9 (no trials were included in the review) and the questions related to meta-analysis (i.e., 11, 12, 15). Among the remaining questions, we assigned a score of 1 to each “yes” response and a score of 0.5 to each “partial yes” response, resulting in a maximum score of 13.

### Departures from the protocol

We assessed risk of bias using AXIS because all of the included articles were cross-sectional. We added AMSTAR 2 to assess the methodological quality of the systematic review.

## Results

A total of 1391 records were retrieved for both research questions. Four eligible articles (two per research question) were ultimately included in the systematic review. Detailed results are discussed below. Examples of excluded articles would be “Satisfaction with rollators among community-living users: a follow-up study”, which examined AD yet did not measure LS and “We have built it, but they have not come: Examining the adoption and use of assistive technologies for informal family caregivers” that did not investigate caregiving hours [49, 50].

For Research Question 1, a total of 963 citations were retrieved by searching the databases CINAHL, MEDLINE, and Scopus. After removing 47 duplicates, 916 proceeded to title and abstract screening. Seven hundred and sixty-four (83%) articles were removed during the title and abstract screening, leaving 152 articles for full-text screening. One hundred and fifty articles were omitted for not meeting the eligibility criteria. A list of excluded studies is available upon request. The systematic review includes two studies that met the eligibility criteria. Searching through reference lists did not yield any additional articles.

**Fig. 1:**
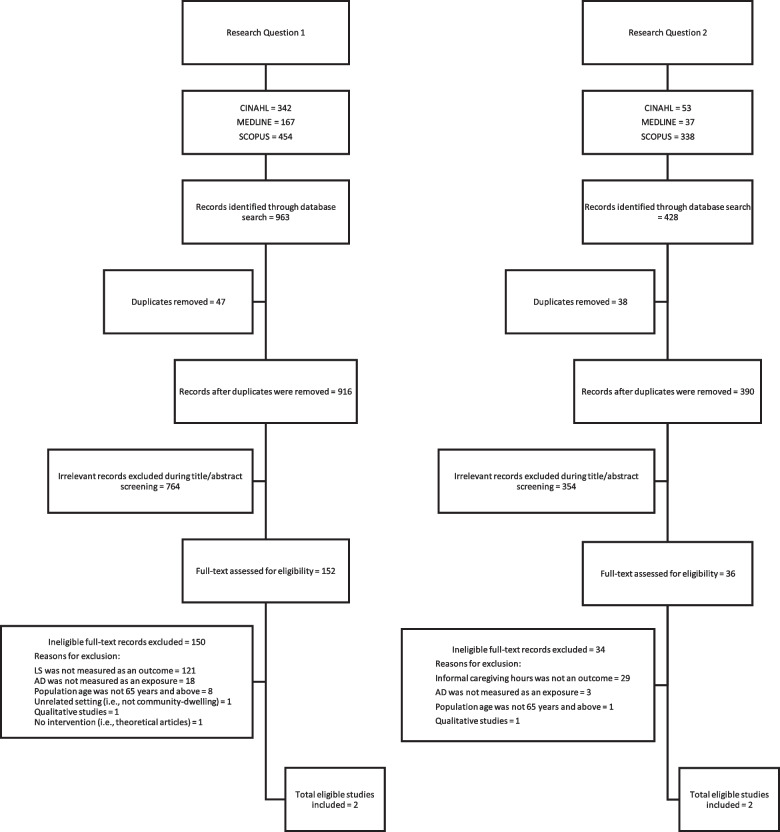
PRISMA flow chart for Research Question 1 and Research Question 2

For Research Question 2, a total of 428 articles were retrieved by searching the same databases as in Research Question 1. After removing 38 duplicates, 390 studies remained eligible for title and abstract screening. Three hundred and fifty-four (91%) studies were removed during title and abstract screening and 36 studies advanced to full text screening. Thirty-four studies were excluded because they did not meet the eligibility criteria. A complete list of excluded studies is available upon request from the authors. Two studies met the eligibility criteria and were included in the systematic review. Figure 1: PRISMA flow chart for Research Question 1 and Research Question 2, depicts the flow of articles through the screening process.

### Study characteristics

#### Research question 1: assistive device use and life satisfaction

The two eligible articles were cross-sectional [51, 52]. One article was published in English and the other in Korean, which was translated with the assistance of a native Korean speaker. The two articles defined the outcome, LS, as an individual’s subjective experience of fulfillment in life or one’s global degree of contentment with life [51, 52], and measured with the Elderly LS Scale and the Andrew and Withey LS Scale adapted by Alex Michalos [51, 52]. Both articles reported on community-dwelling adults aged 65 years and older and treated the exposure (AD use) as dichotomous (AD use versus no use).

The first article, by In-sook et al., investigated the association between the use of devices for walking, bathing, daily living, healthcare, physiotherapy, and health monitoring among 601 community-dwelling, older Korean adults [51]. After adjusting for age, income, gender, marital status, living arrangements, subjective health, activities of daily living and instrumental activities of daily living (ADLs/IADLs), health related quality of life, and satisfaction with AD, the authors reported no association between the use of AD and LS ($\hat{\upbeta}$ = 0.014 – not significant; note the authors did not report a *p*-value or standard error).

The second article, by Leung et al., used data from the Canadian Study for Health and Aging (CSHA) to investigate whether wheelchair use was associated with LS in a group of 5395 community-dwelling persons aged 65 years or older who were not diagnosed with dementia [52]. The authors reported that the rating of life as a whole among persons with a wheelchair was lower than that of persons without a wheelchair (X^2^ = 68.5, *p* < 0.0001), suggesting older adults using wheelchairs experienced less satisfaction with life compared to those who did not use wheelchairs. However, this paper did not report any results besides the chi-square test statistic and *p*-value.

#### Research question 2: assistive device use and informal caregiving hours

The two included articles for Research Question 2 were cross-sectional and published in English [53, 54]. Both articles defined informal care similarly: that is, unpaid care provided by family and friends. Both articles measured time spent on informal caregiving in hours as the outcome measure. One article collected data on informal caregiving hours received in the week before data collection [54], while the other looked at informal caregiving hours received in the two weeks prior to data collection [53]. In Agree et al.’s study, respondents were asked how many numbers of hours of hands-on assistance they received for ADL activities [53]. Hoenig et al. asked respondents how many hours of assistance they received to perform basic ADLs (i.e., eating, getting in and out of bed, getting around inside, dressing, bathing, and getting to the bathroom or toilet) [54]. Both articles obtained data on informal caregiving hours from the care recipients [53, 54]. The common exposure investigated by both articles was AD use (versus non-use) and the samples included community-dwelling adults aged 65 years or older.

Agree et al. investigated whether the use of ADs for mobility (i.e., cane, walker, wheelchair), bathing (i.e., bath seat and rail), or toileting (i.e., raised seat, toilet rail, portable toilet) was associated with reductions in informal caregiving hours among 4006 community-dwelling, older American adults, who had any pathology, impairment limitation, or disability [53]. Their study used the method of multivariate regression analysis, where interdependent outcomes of AD use, formal care, and informal care were simultaneously regressed on variables covering health needs, resources, access, and demographic characteristics. Using this methodology, the authors identified factors (independent variables) that were associated with increased likelihood of AD use, while simultaneously associated with decreases in hours used in informal or formal care. Specifically, AD use was significantly associated with fewer informal care hours, particularly among the unmarried (AD use: $\hat{\upbeta}$ = 0.14, *p* < 0.01; Informal care hours: $\hat{\upbeta}$ = − 40.17, p < 0.01), better educated (AD use: $\hat{\upbeta}$ = 0.12, *p* < 0.05; Informal care hours: $\hat{\upbeta}$ = − 15.36, p < 0.01) or had better cognitive abilities (AD use: $\hat{\upbeta}$ = − 0.17, p < 0.01; Informal care hours: $\hat{\upbeta}$ = 83.77, p < 0.01) [53]. These findings adjusted for the number of ADLs that were severely difficult to perform; cognitive impairment measured by the use of a proxy for poor memory; senility and confusion due to having Alzheimer’s disease; insurance; poverty; marital status; living environment; access to healthcare; and demographics such as age, gender, education, and race [53].

In addition, we calculated a small Hedges’ g of 0.22 (95% confidence interval [CI] = 0.15 to 0.28) when comparing informal care hours in persons who utilized AD (*n* = 2712) with those who did not (*n* = 1294), suggesting no difference between the two groups among all participants in the study. Among only respondents who used care, Hedges’ g coefficient for informal care hours among persons with AD and persons without AD was 0.11 (95% CI = 0.04 to 0.17), again indicating a very small difference between the two groups, suggesting no significant difference between groups [53].

The study by Hoenig et al., examined 2638 community-dwelling, American older adults with at least one basic ADL limitation. These individuals reported using any technological aid to help ameliorate ADL impairments in areas such as eating, getting in and out of bed, dressing, bathing, toileting, indoor mobility, and outdoor mobility [54]. After controlling for ADL impairment, missing hours of help, cognitive impairment, health, hospitalizations, age, gender, race, education, income, and insurance, the study found that those who used any technological assistance reported 3.8 fewer hours of help per week ($\hat{\upbeta}$ = − 3.8, 95% CI = − 6.54 to − 1.06) than those who did not [54].

In Research Question 1, one study found an inverse association between AD use and LS, while the other did not find an association. In Research Question 2, one study found a positive association between AD use and reduction in informal caregiving hours received by community-dwelling older adults who have functional impairments, while the other study did not find an association.

#### Risk of Bias: research question 1

In the two studies in Research Question 1, one article scored 16 out of 19 [52], and the other scored 14 (See Additional file 1: APPENDIX F [51]. One article had several limitations associated with the study design (Question 2), justification of results (Question 17), and discussion of limitations (Question 18) [52]. The other article had several limitations associated with justification of sample size (Question 3), addressing non-responders and non-response bias (Questions 7 and 13), and reporting funding sources (Question 19) [51]. Both articles failed to report ethics approval and informed consent processes (Question 20) [51, 52]. In addition, one study did not report any results besides the chi-square test statistic and *p*-value, and also did not account for confounding or provide distributions of results [52]. Overall, the risk of bias was serious across the two studies.

All observational studies were graded as low quality at the start of the GRADE assessment, as per GRADE guidelines. Inconsistency was graded as being serious because one study [51] found no association and the other reported an inverse association between AD use and LS [52]. The results of the latter study should be interpreted with caution because the study did not perform any analysis beyond a chi-square test. All studies directly compared the exposure and outcome of interest. Studies with a large sample size, but only a small number of exposed subjects compared to unexposed subjects, as well as studies with a lack of information on exposed versus non-exposed groups, could potentially be indicative of less precise estimates, as described by Carlson et al. [51, 52, 55]. Confounding was not assessed in both studies as per GRADE guidelines, which state that the impact of plausible confounding should only be assessed in observational studies that have not been downgraded for any reason. The overall quality of evidence of the included studies in Research Question 1 was very low (Table 1).

**Table 1:** GRADE assessment for research question 1

Certainty assessment							Certainty	Importance
№ of studies	Study design	Risk of bias	Inconsistency	Indirectness	Imprecision	Other considerations		
3	observational studies	serious^a^	serious^b^	not serious	serious^c^	none	⨁◯◯◯ Very low	CRITICAL

^a^See Risk of Bias section in results and Additional file 1: APPENDIX F

^b^The results of the two studies were not consistent

^c^The study by Leung et al. [52] had a large sample size (*n* = 5395) and unbalanced exposure groups (exposed = 295, 5.5% vs. unexposed = 4949, 92.5%). The study by In-sook et al. had a medium sample size (*n* = 601); this study did not report the number of persons with AD and persons without AD [51]

#### Risk of Bias: Research Question 2

In the two studies in Research Question 2, both studies scored 18 out of 19 (See Additional file 1: APPENDIX F. One study had a response rate of 51% for the outcome variable and raised concerns about non-response bias (Question 13) [54]. This study also failed to report funding sources [54]. Both studies did not mention if ethics approval was obtained [53, 54]. The risk of bias was not serious across the two studies.

Inconsistency was graded as serious because one study found a positive association between AD use and a reduction in informal caregiving hours [54], whereas the other study reported no association [53]. Indirectness was not serious because both studies directly examined the exposure and outcome of interest. Imprecision was serious due to unbalanced exposure versus non-exposure groups [53, 54]. As per GRADE guidelines mentioned above, plausible confounding was not assessed. The overall quality of evidence of the included studies was low (Table 2).

**Table 2:** GRADE assessment for research question 2

Certainty assessment							Certainty	Importance
№ of studies	Study design	Risk of bias	Inconsistency	Indirectness	Imprecision	Other considerations		
3	observational studies	not serious	serious^a^	not serious	serious^b^	none	⨁⨁◯◯ Low	CRITICAL

^a^The results of the two studies were not consistent

^b^One study had a large sample size (*n* = 2638), but consisted of unbalanced exposure (*n* = 2199, 83.4%) versus non-exposure groups (*n* = 169, 6.41%) [54]. Although the other study had a large sample size, the number of exposed versus unexposed participants was unclear [53]

## Discussion

Of the 1391 citations screened, we found two articles pertaining to each question, for a total of four. In relation to Research Question 1, one study showed no association between AD use and LS and one study found an inverse relationship [51, 52]. In Research Question 2, two studies showed a positive finding for the association between AD use and the reduction in informal caregiving hours [53, 54]. The dearth and limitations of published literature on both research questions prevented us from drawing firm conclusions about the associations under study.

A few limitations of the studies included in this review should be noted. The two studies included in Research Question 1 had inconsistent results, possibly due to heterogeneity across studies. One study adjusted for a wide range of covariates [51] and the other study did not control for any covariates, presenting varying degrees of confounding effects [52]. Additionally, each study investigated the use of different AD (i.e., wheelchairs, and devices for walking, bathing, daily living, healthcare, physiotherapy, and health monitoring), therefore limiting the comparison of findings across studies. Furthermore, the two studies occupied different statistical analyses.

Similar to studies in Research Question 1, the studies in Research Question 2 had discrepancies in the results. The two studies controlled for different mixes of covariates, which may have contributed to differences in the strength and direction of the results across studies. Both sets of study authors acknowledged the potential presence of residual confounding because they did not control for comorbid health conditions [53, 54]. Both studies had further limitations such as concerns around non-response bias and imprecision (See Table 2, footnote ‘b’), further biasing the results. Non-response bias can be suspected when non-responders in a study are different from responders on prognostic characteristics [44]. For example, in the study by Hoenig et al., participants who did not respond to questions relating to ADL impairment or hours of help reported using significantly more hours of help, possibly shifting the results toward the null. One study investigated those who utilized AD for only ADL difficulties; therefore, the findings cannot be generalized to community-dwelling older adults who use other types of AD [53]. The quality of evidence in Research Question 2 research was low, along with only two studies, making it difficult to draw firm conclusions or make recommendations.

A common limitation across studies was that many types of AD were grouped together under “AD use”, thus obscuring the differences between AD in terms of their individual impacts on the two outcomes (LS and informal caregiving hours) [51, 53, 54]. Researchers should stratify their analyses by specific types of AD (e.g., mobility versus hearing-related AD, high-tech versus low-tech AD) when possible, to understand whether device-specific differences exist. Such analyses would require large sample sizes and future research could perhaps power their studies to explore differences between multiple devices. In addition, existing evidence may lack internal validity due to the very low (Research Question 1) and low (Research Question 2) strength of evidence. All four studies were cross-sectional, which prevents inferences about temporality and changing relationships over time. The cross-sectional nature, and the strength evidence of existing studies creates uncertainty around whether the results of existing studies present true associations. Lastly, findings may be only relevant to populations from areas similar to where the studies were carried out (i.e., Canada, South Korea, and United States) and therefore may not be generalizable to all countries.

This systematic review identified important gaps in the literature. Future studies can overcome the aforementioned knowledge gaps by first, conducting further research (i.e., longitudinal, stratified by AD) to overcome the knowledge and methodological gaps. Through attempting to reduce limitations observed in existing studies (i.e., risk of bias, residual confounding, non-response bias), by controlling for appropriate confounding variables, having balanced exposure versus non-exposure groups, and reducing other biases such as those that are mentioned under ‘Risk of Bias’, future studies can improve the quality and strength of evidence, increasing their reliability for decision-making.

## Strengths and limitations of the review

This systematic review is the first to assess the impact of AD on LS and informal caregiving hours received among community-dwelling older adults. Furthermore, this systematic review considered articles that were published in other languages besides English to minimize the possibility of language bias. This review also followed the PRISMA criteria for systematic reviews and utilized GRADE and AXIS for assessing the quality of articles. Finally, a self-rating using AMSTAR 2 scored this review 12 out of 13, indicating high methodological quality. Question 4 and 7 of AMSTAR received a 0.5, or “partial yes”, because we did not search grey literature and trial/study registries and the list of excluded publications was not provided in an appendix due to length.

Limitations of the review included an inability to conduct a meta-analysis due to the heterogeneity of the articles, as well as a lack of commonly-reported outcome statistics. We were also unable to assess publication bias due to the absence of a meta-analysis and the small number of studies included in the review.

## Conclusion

The scarcity of studies and low to very low strength of existing evidence prevented us from drawing conclusions about the two associations investigated in this review. AD play an important role in improving the overall well-being of community-dwelling older adults. This review considers the potential of AD in improving LS of, and reducing informal caregiving hours received by, community-dwelling older adults. Greater LS levels can enhance the state and experience of living as an older adult, while reductions in informal care hours can ease the negative outcomes associated with greater hours of informal caregiving (i.e., caregiver stress, depression). More research and high-quality evidence are required for evidence-based decision-making and effective recommendations regarding the provision and funding of AD for community-dwelling older adults.

## Supplementary Information

**Additional file 1.**

## Data Availability

The datasets used and analysed during the current study are available from the authors on reasonable request.

## Abbreviations

AD: Assistive devices
ADLs: Activities of daily living
AMSTAR: Assessment of multiple systematic reviews
AXIS: Appraisal Tool for Cross-Sectional Studies
CATOR: Consortium for Assistive Technology Outcomes Research
CI: Confidence interval
CSHA: Canadian Study for Health and Aging
GRADE: Grading of Recommendation, Assessment, Development, and Evaluation
IADLs: Instrumental activities of daily living
ISO: International Organization for Standardization
LS: Life satisfaction
PRISMA: Preferred Reporting Items for Systematic reviews and Meta-Analyses
PROSPERO: International Prospective Register of Systematic Reviews
R: R Foundation for Statistical Computing
WHO: World Health Organization

## References

[1] World Health Organization Regional Office for Europe. Assistive technology. https://www.who.int/westernpacific/health-topics/assistive-technology (2021). Accessed 12 Jun 12 2021.

[2] Government of Canada. Seniors and Aging - Assistive Devices. https://www.canada.ca/en/health-canada/services/healthy-living/your-health/lifestyles/seniors-aging-assistive-devices.html (2007). Accessed Jun 12 2021.

[3] Löfqvist C, Nygren C, Széman Z, Iwarsson S (2005). Assistive devices among very old people in five European countries. Scand J Occup Ther.

[4] Roelands M, Van Oost P, Depoorter A, Buysse A (2002). A social-cognitive model to predict the use of assistive devices for mobility and self-care in elderly people. Gerontologist..

[5] Mann WC, Ottenbacher KJ, Fraas L, Tomita M, Granger CV (1999). Effectiveness of assistive technology and environmental interventions in maintaining independence and reducing home care costs for the frail elderly. A randomized controlled trial. Arch Fam Med.

[6] Scott V, Dukeshire S, Gallagher E, Scanlan A. A Best Practices Guide for the Prevention of Falls Among Seniors Living in the Community. Division of Aging and Seniors. 2001. http://www.injuryresearch.bc.ca/docs/3_20061220_105723BestPractice_Falls_e.pdf. Accessed 13 Jun 2021.

[7] Department of Health and Ageing. Comprehensive Scoping Study on the Use of Assistive Technology by Frail Older People Living in the Community. Department of Health and Ageing. 2008. https://apo.org.au/sites/default/files/resource-files/2011-02/apo-nid23803.pdf. Accessed 13 Jun 2021.

[8] Heywood F, Turner L. Implications for health and social care budgets of Investments in Housing Adaptations, Improvements and Equipment: A Review of the Evidence. In Better Outcomes, Lower Costs. Office for Disability Issues. 2007. http://www.officefordisability.gov.uk/docs/better_outcomes_report.pdf. Accessed Jun 12 2021.

[9] McConatha D, McConatha JT, Dermigny R (1994). The use of interactive computer services to enhance the quality of life for long-term care residents. Gerontologist..

[10] Marasinghe KM, Lapitan JM, Ross A (2015). Assistive technologies for ageing populations in six low-income and middle-income countries: a systematic review. BMJ Innov.

[11] United Nations. World Population Ageing 2019 Highlights. United Nations. 2019. https://www.un.org/en/development/desa/population/publications/pdf/ageing/WorldPopulationAgeing2019-Highlights.pdf. Accessed Jun12 2021.

[12] World Health Organization. Global Health and Aging. 2011. https://www.nia.nih.gov/sites/default/files/2017-06/global_health_aging.pdf. Accessed Mar 03 2021.

[13] Foreman KJ, Marquez N, Dolgert A (2018). Forecasting life expectancy, years of life lost, and all-cause and cause-specific mortality for 250 causes of death: reference and alternative scenarios for 2016–40 for 195 countries and territories. Lancet..

[14] Cao X, Hou Y, Zhang X (2020). A comparative, correlate analysis and projection of global and regional life expectancy, healthy life expectancy, and their GAP: 1995-2025. J Glob Health..

[15] Diener E, Emmons RA, Larsen RJ, Griffin S (1985). The satisfaction with life scale. J Pers Assess.

[16] OECD. How’s Life? Measuring Well-being. https://www.oecd-ilibrary.org/economics/how-s-life_9789264121164-en (2011). Accessed Jun 12 2021.

[17] Diener E, Chan MY (2011). Happy people live longer: subjective well-being contributes to health and longevity. Appl Psychol Health Well-Being.

[18] Rosella LC, Fu L, Buajitti E, Goel V (2019). Death and chronic disease risk associated with poor life satisfaction: a population-based cohort study. Am J Epidemiol.

[19] World Health Organization Regional Office for Europe. Life Satisfaction. https://gateway.euro.who.int/en/indicators/h2020_24-life-satisfaction/ (2020). Accessed Jun 12 2021.

[20] Government of Canada. An Assessment of Life Satisfaction Responses on Recent Statistics Canada Surveys: Main article. https://www150.statcan.gc.ca/n1/pub/11f0019m/2013351/part-partie1-eng.htm (2015). Accessed Jun 12 2021.

[21] Williams GM (2020). Life satisfaction and associated predictors in an older adult population. M.Sc.

[22] World Health Organization. WHOQOL User Manual. https://apps.who.int/iris/bitstream/handle/10665/77932/WHO_HIS_HSI_Rev.2012.03_eng.pdf;jsessionid=890F1A47210937A5DC04503E83BB6313?sequence=1 (1998). Accessed Jun 12 2021.

[23] Massey B, Edwards A, Musikanski L (2021). Life satisfaction, affect, and belonging in older adults. Appl Res Qual Life.

[24] Kim ES, Kubzansky LD, Smith J (2015). Life satisfaction and use of preventive health care services. Health Psychol.

[25] Kim E, Delaney S, Tay L, Chen Y, Diener E, Vanderweele T (2021). Life satisfaction and subsequent physical, behavioral, and psychosocial health in older adults. Milbank Q.

[26] Jutai JW, Fuhrer MJ, Demers L, Scherer MJ, DeRuyter F (2005). Toward a taxonomy of assistive technology device outcomes. Am J Phys Med Rehabil.

[27] Canadian Longitudinal Study on Aging. CLSA 60-min. Questionnaire (Tracking Main Wave). 2018. https://www.clsa-elcv.ca/data-collection. Accessed Jan 04 2022.

[28] MacDonald BJ, Wolfson M, Hirdes J. The future co$t of long-term Care in Canada. 2019. https://static1.squarespace.com/static/5c2fa7b03917eed9b5a436d8/t/5dbadf6ce6598c340ee6978f/1572527988847/The+Future+Cost+of+Long-Term+Care+in+Canada.pdf. Accessed Jan 20 2022.

[29] Mortensen J, Dich N, Lange T (2018). Weekly hours of informal caregiving and paid work, and the risk of cardiovascular disease. Eur J Pub Health.

[30] Seniors Advocate British Columbia. Caregivers in distress - a growing problem. 2017. https://www.seniorsadvocatebc.ca/app/uploads/sites/4/2017/08/Caregivers-in-Distress-A-Growing-Problem-Final.pdf. Accessed Jan 22 2022.

[31] Lindt N, van Berkel J, Mulder B (2020). Determinants of overburdening among informal carers: a systematic review. BMC Geriatr.

[32] Government of Canada SC. The Daily - Differences in the characteristics of caregivers and caregiving arrangements of Canadians, 2018. 2022. https://www150.statcan.gc.ca/n1/daily-quotidien/220114/dq220114c-eng.htm. Accessed Jan 18 2022.

[33] Elmståhl S, Lundholm-Auoja N, Ekström H, Sandin WL (2020). Being an older family caregiver does not impact healthcare and mortality: Data from the study ‘Good Aging in Skåne.’. Scand J Public Health.

[34] Konerding U, Bowen T, Forte P (2019). Do caregiver characteristics affect caregiver burden differently in different countries?. Am J Alzheimers Dis Other Dement.

[35] Prevo L, Hajema K, Linssen E, Kremers S, Crutzen R, Schneider F. Population characteristics and needs of informal caregivers associated with the risk of perceiving a high burden: a cross-sectional study. Inquiry. 2018;55. 10.1177/0046958018775570. 10.1177/0046958018775570 PMC5977419 29808748

[36] Turner A, Findlay L (2012). Informal caregiving for seniors. Health Rep.

[37] Ysseldyk R, Kuran N, Powell S, Villeneuve PJ (2019). Original quantitative research self-reported health impacts of caregiving by age and income among participants of the Canadian 2012 general social survey. Health Promot Chronic Dis Prev Can.

[38] Fredman L, Cauley JA, Satterfield S (2008). Caregiving and risk of mortality and functional decline in white and black elderly adults: findings from the health ABC study. Arch Intern Med.

[39] Loi SM, Dow B, Moore K (2016). Factors associated with depression in older carers. Int J Geriatr Psychiatry.

[40] Oliveira DC, Vass C, Aubeeluck A (2018). The development and validation of the dementia quality of life scale for older family Carers (DQoL-OC). Aging Ment Health.

[41] Carers UK, Age UK. Caring into later life - the growing pressure on older carers. 2015. https://www.carersuk.org/component/cck/?task=download&collection=file_list&xi=0&file=document&id=5224. Accessed Jan 25 2022.

[42] Tuazon JR, Jutai JW (2021). Toward guidelines for reporting assistive technology device outcomes. Disabil Rehabil Assist Technol.

[43] Liberati A, Altman DG, Tetzlaff J (2009). The PRISMA statement for reporting systematic reviews and meta-analyses of studies that evaluate healthcare interventions: explanation and elaboration. BMJ..

[44] Appraisal tool for Cross-Sectional Studies (AXIS). N.d. https://bmjopen.bmj.com/content/bmjopen/6/12/e011458/DC2/embed/inline-supplementary-material-2.pdf?download=true. Accessed Mar 13 2022.

[45] Wong JN, McAuley E, Trinh L (2018). Physical activity programming and counseling preferences among cancer survivors: a systematic review. Int J Behav Nutr Phys Act.

[46] Moskalewicz A, Oremus M (2020). No clear choice between Newcastle–Ottawa scale and appraisal tool for cross-sectional studies to assess methodological quality in cross-sectional studies of health-related quality of life and breast cancer. J Clin Epidemiol.

[47] GRADE handbook. 2013. https://gdt.gradepro.org/app/handbook/handbook.html#h.xivvyiu1pr3v. Accessed Jun 28 2021.

[48] AMSTAR 2 - assessing the methodological quality of systematic reviews. 2021. https://amstar.ca/Amstar_Checklist.php. Accessed Mar 15 2022.

[49] Brandt A, Iwarsson S, Stahl A (2003). Satisfaction with rollators among community-living users: a follow-up study. Disabil Rehabil.

[50] Wisniewski P, Linton C, Chokshi A, Perlingieri B, Gurupur V, Gabriel M. We have built it, but they have not come: examining the adoption and use of assistive Technologies for Informal Family Caregivers. In: Ahram TZ, Falcão C, editors. Advances in usability, user experience and assistive technology. Advances in intelligent systems and computing. Cham: Springer International Publishing; 2019. p. 824–36.

[51] Lee I, Kim J (2008). The effect of assistive devices and appliances on life satisfaction among the elderly. Social Welfare Policy.

[52] Leung V, Colantonio A, Santaguida PL (2005). Wheelchair use, pain, and satisfaction with life in a national sample of older adults. Gerontechnology..

[53] Agree EM, Freedman VA, Cornman JC, Wolf DA, Marcotte JE (2005). Reconsidering substitution in long-term care: when does assistive technology take the place of personal care?. J Gerontol B Psycholo Sci Soc Sci.

[54] Hoenig H, Taylor DH, Sloan FA (2003). Does assistive technology substitute for personal assistance among the disabled elderly?. Am J Public Health.

[55] Carlson MDA, Morrison RS (2009). Study design, precision, and validity in observational studies. J Palliat Med.

