# Correction: The impact of assistive devices on community-dwelling older adults and their informal caregivers: a systematic review

**DOI:** 10.1186/s12877-023-03888-0

**Published:** 2023-04-04

**Authors:** Keshini Madara Marasinghe, Ashok Chaurasia, Maisha Adil, Qian Yue Liu, Teeyaa Ibrahim Nur, Mark Oremus

**Affiliations:** 1 School of Public Health Sciences, University of Waterloo, 200 University Avenue West, Waterloo, ON Canada; 2 Oxford Institute of Population Ageing, University of Oxford, Oxford, UK; 3 UBC Hospital, 2211 Wesbrook Mall, Vancouver, BC Canada

**Correction: BMC Geriatrics 22, 897 (2022)**

**https://doi.org/10.1186/s12877-022-03557-8**

After publication of this article [1], the authors detected errors in the reporting of the results and quality assessment.

The last sentence of “Research question 2: assistive device use and informal caregiving hours” should read as: “In Research Question 2, both studies found a positive association between AD use and reduction in informal caregiving hours received by community-dwelling older adults.”

The first sentence of the last paragraph of “Risk of Bias: research question 2” should read as: “Inconsistency was graded as not serious because both studies found a positive association between AD use and a reduction in informal caregiving hours [53, 54].”

The number in the column, “№ of studies” in Table 1, should read as 2.

Table 2 should read as:

**Table:** 

Certainty assessment								
№ of studies	Study design	Risk of bias	Inconsistency	Indirectness	Imprecision	Other considerations	Certainty	Importance
2	observational studies	not serious	not serious	not serious	serious^a^	none	⊕⊕⊕◯ Moderate	CRITICAL

^a^One study had a large sample size (n = 2638), but consisted of unbalanced exposure (n = 2199, 83.4%) versus non-exposure groups (n = 169, 6.41%) [54]. Although the other study had a large sample size, the number of exposed versus unexposed participants was unclear [53].

The above update to Table 2 changed the overall quality of evidence in Research Question 2, from “low” to “moderate” and this change should be updated in the following sections as shown.

Abstract

Results (last sentence) and Conclusion (first sentence).

Main body of the article.

Risk of Bias: research question 2 (last paragraph, last sentence).

Discussion (third paragraph, last sentence; and fourth paragraph, fourth sentence).

Conclusion (first sentence).

In the Discussion section, the first sentence of the third paragraph should be removed. The next sentence should read as: “In Research Question 2, the two studies controlled for different mixes of covariates, which may have contributed to differences in the strength of the results across studies.”
