# Supplementary material for: The impact of assistive devices on community-dwelling older adults and their informal caregivers: a systematic review
Source: BMC Geriatr. 2022 Nov 24;22:897. doi: 10.1186/s12877-022-03557-8 (PMC9686024; doi:10.1186/s12877-022-03557-8)
Supplement: Supplementary file 1 — Additional file 1. [file 12877_2022_3557_MOESM1_ESM.docx]

# ADDITIONAL FILE 1

## APPENDIX A

PRISMA checklist for the abstract.

| **Section and Topic** | **Item #** | **Checklist item** | **Reported (Yes/No)** |
| --- | --- | --- | --- |
| **TITLE** | | |  |
| Title | 1 | Identify the report as a systematic review. | Yes |
| **BACKGROUND** | | |  |
| Objectives | 2 | Provide an explicit statement of the main objective(s) or question(s) the review addresses. | Yes |
| **METHODS** | | |  |
| Eligibility criteria | 3 | Specify the inclusion and exclusion criteria for the review. | No. Mentioned elsewhere. |
| Information sources | 4 | Specify the information sources (e.g., databases, registers) used to identify studies and the date when each was last searched. | Yes |
| Risk of bias | 5 | Specify the methods used to assess risk of bias in the included studies. | No. Mentioned elsewhere. |
| Synthesis of results | 6 | Specify the methods used to present and synthesise results. | Yes |
| **RESULTS** | | |  |
| Included studies | 7 | Give the total number of included studies and participants and summarise relevant characteristics of studies. | Yes |
| Synthesis of results | 8 | Present results for main outcomes, preferably indicating the number of included studies and participants for each. If meta-analysis was done, report the summary estimate and confidence/credible interval. If comparing groups, indicate the direction of the effect (i.e., which group is favoured). | Yes. Number of participants not mentioned. |
| **DISCUSSION** | | |  |
| Limitations of evidence | 9 | Provide a brief summary of the limitations of the evidence included in the review (e.g., study risk of bias, inconsistency, and imprecision). | Yes |
| Interpretation | 10 | Provide a general interpretation of the results and important implications. | Yes |
| **OTHER** | | |  |
| Funding | 11 | Specify the primary source of funding for the review. | No. Mentioned elsewhere. |
| Registration | 12 | Provide the register name and registration number. | Yes |

From:  Page MJ, McKenzie JE, Bossuyt PM, Boutron I, Hoffmann TC, Mulrow CD, et al. The PRISMA 2020 statement: an updated guideline for reporting systematic reviews. BMJ 2021;372:n71. doi: 10.1136/bmj.n71

PRISMA Checklist for the Article

| **Section and Topic** | **Item #** | **Checklist item** | **Location where item is reported** |
| --- | --- | --- | --- |
| **TITLE** | | |  |
| Title | 1 | Identify the report as a systematic review. | 1 |
| **ABSTRACT** | | |  |
| Abstract | 2 | See the PRISMA 2020 for Abstracts checklist. | N/A |
| **INTRODUCTION** | | |  |
| Rationale | 3 | Describe the rationale for the review in the context of existing knowledge. | 5-7 |
| Objectives | 4 | Provide an explicit statement of the objective(s) or question(s) the review addresses. | 7 |
| **METHODS** | | |  |
| Eligibility criteria | 5 | Specify the inclusion and exclusion criteria for the review and how studies were grouped for the syntheses. | 8 |
| Information sources | 6 | Specify all databases, registers, websites, organisations, reference lists and other sources searched or consulted to identify studies. Specify the date when each source was last searched or consulted. | 7 |
| Search strategy | 7 | Present the full search strategies for all databases, registers and websites, including any filters and limits used. | 7 |
| Selection process | 8 | Specify the methods used to decide whether a study met the inclusion criteria of the review, including how many reviewers screened each record and each report retrieved, whether they worked independently, and if applicable, details of automation tools used in the process. | 8 |
| Data collection process | 9 | Specify the methods used to collect data from reports, including how many reviewers collected data from each report, whether they worked independently, any processes for obtaining or confirming data from study investigators, and if applicable, details of automation tools used in the process. | 7-8 |
| Data items | 10a | List and define all outcomes for which data were sought. Specify whether all results that were compatible with each outcome domain in each study were sought (e.g., for all measures, time points, analyses), and if not, the methods used to decide which results to collect. | 7-8 |
|  | 10b | List and define all other variables for which data were sought (e.g., participant and intervention characteristics, funding sources). Describe any assumptions made about any missing or unclear information. | 7-8 |
| Study risk of bias assessment | 11 | Specify the methods used to assess risk of bias in the included studies, including details of the tool(s) used, how many reviewers assessed each study and whether they worked independently, and if applicable, details of automation tools used in the process. | 9 |
| Effect measures | 12 | Specify for each outcome the effect measure(s) (e.g., risk ratio, mean difference) used in the synthesis or presentation of results. | 8-9 |
| Synthesis methods | 13a | Describe the processes used to decide which studies were eligible for each synthesis (e.g., tabulating the study intervention characteristics and comparing against the planned groups for each synthesis (item #5)). | 8 |
|  | 13b | Describe any methods required to prepare the data for presentation or synthesis, such as handling of missing summary statistics, or data conversions. | N/A |
|  | 13c | Describe any methods used to tabulate or visually display results of individual studies and syntheses. | 8-9 |
|  | 13d | Describe any methods used to synthesize results and provide a rationale for the choice(s). If meta-analysis was performed, describe the model(s), method(s) to identify the presence and extent of statistical heterogeneity, and software package(s) used. | 9 |
|  | 13e | Describe any methods used to explore possible causes of heterogeneity among study results (e.g., subgroup analysis, meta-regression). | N/A |
|  | 13f | Describe any sensitivity analyses conducted to assess robustness of the synthesized results. | N/A |
| Reporting bias assessment | 14 | Describe any methods used to assess risk of bias due to missing results in a synthesis (arising from reporting biases). | 9 |
| Certainty assessment | 15 | Describe any methods used to assess certainty (or confidence) in the body of evidence for an outcome. | 9 |
| **RESULTS** | | |  |
| Study selection | 16a | Describe the results of the search and selection process, from the number of records identified in the search to the number of studies included in the review, ideally using a flow diagram. | 10-11 |
|  | 16b | Cite studies that might appear to meet the inclusion criteria, but which were excluded, and explain why they were excluded. | N/A |
| Study characteristics | 17 | Cite each included study and present its characteristics. | 11-14 |
| Risk of bias in studies | 18 | Present assessments of risk of bias for each included study. | 14-16 |
| Results of individual studies | 19 | For all outcomes, present, for each study: (a) summary statistics for each group (where appropriate) and (b) an effect estimate and its precision (e.g., confidence/credible interval), ideally using structured tables or plots. | 11-14 |
| Results of syntheses | 20a | For each synthesis, briefly summarise the characteristics and risk of bias among contributing studies. | 11-16 |
|  | 20b | Present results of all statistical syntheses conducted. If meta-analysis was done, present for each the summary estimate and its precision (e.g., confidence/credible interval) and measures of statistical heterogeneity. If comparing groups, describe the direction of the effect. | 13 |
|  | 20c | Present results of all investigations of possible causes of heterogeneity among study results. | 14-16 |
|  | 20d | Present results of all sensitivity analyses conducted to assess the robustness of the synthesized results. | N/A |
| Reporting biases | 21 | Present assessments of risk of bias due to missing results (arising from reporting biases) for each synthesis assessed. | 14-16 |
| Certainty of evidence | 22 | Present assessments of certainty (or confidence) in the body of evidence for each outcome assessed. | 14-16 |
| **DISCUSSION** | | |  |
| Discussion | 23a | Provide a general interpretation of the results in the context of other evidence. | 16-17 |
|  | 23b | Discuss any limitations of the evidence included in the review. | 17-18 |
|  | 23c | Discuss any limitations of the review processes used. | 19 |
|  | 23d | Discuss implications of the results for practice, policy, and future research. | 19-20 |
| **OTHER INFORMATION** | | |  |
| Registration and protocol | 24a | Provide registration information for the review, including register name and registration number, or state that the review was not registered. | 3,7 |
|  | 24b | Indicate where the review protocol can be accessed, or state that a protocol was not prepared. | 7 |
|  | 24c | Describe and explain any amendments to information provided at registration or in the protocol. | 10 |
| Support | 25 | Describe sources of financial or non-financial support for the review, and the role of the funders or sponsors in the review. | 22 |
| Competing interests | 26 | Declare any competing interests of review authors. | 22 |
| Availability of data, code and other materials | 27 | Report which of the following are publicly available and where they can be found: template data collection forms; data extracted from included studies; data used for all analyses; analytic code; any other materials used in the review. | N/A |

From:  Page MJ, McKenzie JE, Bossuyt PM, Boutron I, Hoffmann TC, Mulrow CD, et al. The PRISMA 2020 statement: an updated guideline for reporting systematic reviews. BMJ 2021;372:n71. doi: 10.1136/bmj.n71

## APPENDIX B

Search terms and results for Research Question 1

|  | Scopus | PubMed | CINAHL |
| --- | --- | --- | --- |
| ( TITLE-ABS-KEY ( ( “assistive device*” AND mobility ) OR “assistive technology*” OR “assistive device*” OR “assistive product*” OR gerontechnolog* OR “self-help device*” OR “self-help device*” OR “selfhood device” OR “home modification*” OR “mobility aid*” OR “prosth*” OR “ortho*” OR wheelchair* OR cane* OR stick* OR seat* OR walker* OR “walking frame*” OR scooter* OR rollator* OR chair* OR bath* OR detector* OR gps OR “supportive device*” OR “brace*” OR crutch* OR “motorized scooter*” OR “hip protector*” OR “mobility device*” OR “mobility product*” OR “grab bar*” OR rail* OR ramp* OR “bathroom aid*” OR “bath lift*” OR “bed lift*” OR “lifting device*” OR “lifting product*” OR “lifting technolog*” OR “grasping tool*” OR “reach extender*” OR “eating utensil*” OR “personal alarm*” OR “pencil grip*” OR “pill organizer*” OR footwear* OR tricycle* OR seat* OR toilet* OR bathroom* OR cushion* OR pillow* OR bed* OR shower* OR door* OR ( device AND dressing* ) OR doorway* OR hallway* OR electrotherapy OR ( “assistive device*” AND pen ) OR ( “assistive device*” AND print ) OR ( “assistive device*” AND hearing ) OR “hearing aid*” OR “cochlear implant*” OR amplifier* OR “volume control telephone” OR “TTY” OR teletype OR “TTD” OR “telecommunications device for the deaf” OR “text telephone*” OR “message relay service*” OR ( “hearing AND loop” ) OR flasher* OR decoder* OR “closed caption” OR “closed caption television” OR “fm amplifier*” OR ( “assistive device*” AND “infra-red” ) OR ( “assistive device*” AND infrared ) OR acoustic OR “visual alarm*” OR “vibrating alarm*” OR “closed caption*” OR software* OR ( “assistive device*” AND vision ) OR magnifier* OR braille OR “talking book*” OR “portable note-taker*” OR “screen reader*” OR “voice recognition” OR daisy OR “communication software*” OR spectacle* OR glass* OR “contact lens*” ) AND TITLE-ABS-KEY ( “older adult*” OR “aging population*” OR elder* OR senior* OR “old people” OR “older people” OR aged OR “frail elder*” ) AND TITLE-ABS-KEY ( “satisfaction of life” OR “life satisfaction” OR “self-satisfaction” OR “satisfaction with life” ) ) | 454 |  |  |
| “Self-Help Devices” AND (Aged OR “Frail Elderly”) AND satisfaction |  | 167 |  |
| AB ( “assistive device*” AND mobility ) OR “assistive technolog*” OR “assistive device*” OR “assistive product*” OR gerontechnolog* OR “self-help device*” OR “self help device*” OR “selfhood device” OR “home modification*” OR “mobility aid*” OR “prosth*” OR “ortho*” OR wheelchair* OR cane* OR stick* OR seat* OR walker* OR “walking frame*” OR scooter* OR rollator* OR chair* OR bath* OR detector* OR gps OR “supportive device*” OR “brace*” OR crutch* OR “motorized scooter*” OR “hip protector*” OR “mobility device*” OR “mobility product*” OR “grab bar*” OR rail* OR ramp* OR “bathroom aid*” OR “bath lift*” OR “bed lift*” OR “lifting device*” OR “lifting product*” OR “lifting technolog*” OR “grasping tool*” OR “reach extender*” OR “eating utensil*” OR “personal alarm*” OR “pencil grip*” OR “pill organizer*” OR footwear* OR tricycle* OR seat* OR toilet* OR bathroom* OR cushion* OR pillow* OR bed* OR shower* OR door* OR ( device AND dressing* ) OR doorway* OR hallway* OR electrotherapy OR ( “assistive device*” AND pen ) OR ( “assistive device*” AND print ) OR ( “assistive device*” AND hearing ) OR “hearing aid*” OR “cochlear implant*” OR amplifier* OR “volume control telephone” OR “TTY” OR teletype OR “TTD” OR “telecommunications device for the deaf” OR “text telephone*” OR “message relay service*” OR ( “hearing AND loop” ) OR flasher* OR decoder* OR “closed caption” OR “closed caption television” OR “fm amplifier*” OR ( “assistive device*” AND “infra-red” ) OR ( “assistive device*” AND infrared ) OR acoustic OR “visual alarm*” OR “vibrating alarm*” OR “closed caption*” OR software* OR ( “assistive device*” AND vision ) OR magnifier* OR braille OR “talking book*” OR “portable note-taker*” OR “screen reader*” OR “voice recognition” OR daisy OR “communication software*” OR spectacle* OR glass* OR “contact lens*” ) AND AB ( “older adult*” OR “aging population*” OR elder* OR senior* OR “old people” OR “older people” OR aged OR “frail elder*” )  AND AB ( “satisfaction of life” OR “life satisfaction” OR “self-satisfaction” OR “satisfaction with life” ) ) |  |  | 342 |
| Total | 963 | | |

Search terms and results for Research Question 2

|  | Scopus | PubMed | CINAHL |
| --- | --- | --- | --- |
| TITLE-ABS-KEY ( ( "assistive device*"  AND  mobility )  OR  "assistive technolog*"  OR  "assistive device*"  OR  "assistive product*"   OR  gerontechnolog*  OR  "self-help device*"  OR  "self help device*"  OR  "selfhood device"  OR  "home modification*"  OR  "mobility aid*"  OR  "prosth*"  OR  "ortho*"  OR  wheelchair*  OR  cane*  OR  stick*  OR  seat*  OR  walker*  OR  "walking frame*"  OR  scooter*  OR  rollator*  OR  chair*  OR  bath*  OR  detector*  OR  gps  OR  "supportive device*"   OR  "brace*"  OR  crutch*  OR  "motorized scooter*"  OR  "hip protector*"  OR  "mobility device*"  OR  "mobility product*"  OR  "grab bar*"  OR  rail*  OR  ramp*  OR  "bathroom aid*"  OR  "bath lift*"  OR  "bed lift*"  OR  "lifting device*"  OR  "lifting product*"  OR  "lifting technolog*"  OR  "grasping tool*"  OR  "reach extender*"  OR  "eating utensil*"   OR  "personal alarm*"  OR  "pencil grip*"  OR  "pill organizer*"  OR  footwear*  OR  tricycle*  OR  seat*  OR  toilet*  OR  bathroom*  OR  cushion*  OR  pillow*  OR  bed*  OR  shower*  OR  door*  OR  ( device  AND  dressing* )  OR  doorway*  OR  hallway*  OR  electrotherapy  OR  ( "assistive device*"  AND  pen )  OR  ( "assistive device*"  AND  print )  OR  ( "assistive device*"  AND  hearing )  OR  "hearing aid*"  OR  "cochlear implant*"  OR  amplifier*  OR  "volume control telephone"  OR  "TTY"  OR  teletype  OR  "TTD"  OR  "telecommunications device for the deaf"  OR  "text telephone*"  OR  "message relay service*"  OR  ( "hearing AND loop" )  OR  flasher*  OR  decoder*  OR  "closed caption"  OR  "closed caption television"  OR  "fm amplifier*"  OR  ( "assistive device*"  AND    "infra-red" )  OR  ( "assistive device*"  AND  infrared )  OR  acoustic  OR  "visual alarm*"   OR  "vibrating alarm*"  OR  "closed caption*"   OR  software*  OR  ( "assistive device*"  AND  vision )  OR  magnifier*  OR  braille  OR  "talking book*"  OR  "portable note-taker*"  OR  "screen reader*"  OR  "voice recognition"  OR  daisy  OR  "communication software*"  OR  spectacle*    OR  glass*  OR  "contact lens*" )  AND  TITLE-ABS-KEY ( "older adult*"  OR  "aging population*"  OR  elder*  OR  senior*  OR  "old people"  OR  "older people"  OR  aged  OR  "frail elder*" )  AND  TITLE-ABS-KEY (informal    OR  "informal care"  OR  "informal caregiv*"  OR  "caregiver burden" )   AND  TITLE-ABS-KEY ( hour*  OR  time ) | 338 |  |  |
| (("Self-Help Devices") AND Caregivers AND (Aged OR "Frail Elderly") AND (hour* OR time OR "time spent")) |  | 37 |  |
| AB ( ( "assistive device*" AND mobility ) OR "assistive technolog*" OR "assistive device*" OR "assistive product*" OR gerontechnolog* OR "self-help device*" OR "self help device*" OR "selfhood device" OR "home modification*" OR "mobility aid*" OR "prosth*" OR "ortho*" OR wheelchair* OR cane* OR stick* OR seat* OR walker* OR "walking frame*" OR scooter* OR rollator* OR chair* OR bath* OR detector* OR gps OR "supportive device*" OR "brace*" OR crutch* OR "motorized scooter*" OR "hip protector*" OR "mobility device*" OR "mobility product*" OR "grab bar*" OR rail* OR ramp* OR "bathroom aid*" OR "bath lift*" OR "bed lift*" OR "lifting device*" OR "lifting product*" OR "lifting technolog*" OR "grasping tool*" OR "reach extender*" OR "eating utensil*" OR "personal alarm*" OR "pencil grip*" OR "pill organizer*" OR footwear* OR tricycle* OR seat* OR toilet* OR bathroom* OR cushion* OR pillow* OR bed* OR shower* OR door* OR ( device AND dressing* ) OR doorway* OR hallway* OR electrotherapy OR ( "assistive device*" AND pen ) OR ( "assistive device*" AND print ) OR ( "assistive device*" AND hearing ) OR "hearing aid*" OR "cochlear implant*" OR amplifier* OR "volume control telephone" OR "TTY" OR teletype OR "TTD" OR "telecommunications device for the deaf" OR "text telephone*" OR "message relay service*" OR ( "hearing AND loop" ) OR flasher* OR decoder* OR "closed caption" OR "closed caption television" OR "fm amplifier*" OR ( "assistive device*" AND "infra-red" ) OR ( "assistive device*" AND infrared ) OR acoustic OR "visual alarm*" OR "vibrating alarm*" OR "closed caption*" OR software* OR ( "assistive device*" AND vision ) OR magnifier* OR braille OR "talking book*" OR "portable note-taker*" OR "screen reader*" OR "voice recognition" OR daisy OR "communication software*" OR spectacle* OR glass* OR "contact lens*") ) AND AB ( "Older adult*" OR "Aging population*" OR Elder* OR Senior* OR "Old people" OR "Older people" OR Aged OR "frail elder*" ) AND AB ( Informal OR "Informal care" OR "Informal caregiv*" OR "caregiver burden" ) AND AB ( hour* OR time ) |  |  | 53 |
| Total | 528 | | |

## APPENDIX C

Classes of assistive devices classified by the ISO 9999 in collaboration with the WHO.

| **ISO 9999 (2016) – 12 classes** | |
| --- | --- |
| 04 | Assistive products for measuring, supporting, training, or replacing body functions |
| 05 | Assistive products for education and for training in skills |
| 06 | Assistive products attached to the body for supporting neuromusculoskeletal or movement related functions (orthoses) and replacing anatomical structures (prostheses) |
| 09 | Assistive products for selfcare activities and participation in selfcare |
| 12 | Assistive products for activities and participation relating to personal mobility and transportation |
| 15 | Assistive products for domestic activities and participation in domestic life |
| 18 | Furnishings, fixtures, and other assistive products for supporting activities in the indoor and outdoor human-made environment |
| 22 | Assistive products for communication and information management |
| 24 | Assistive products for controlling, carrying, moving, and handling objects and devices |
| 27 | Assistive products for controlling, adapting, or measuring elements of the physical environment |
| 28 | Assistive products for work activities and participation in employment |
| 30 | Assistive products for recreation and leisure |

## APPENDIX D

Data extraction table for Research Question 1

| Table 1: Characteristics of eligible studies for Question 1, AD and LS | | | | | | |
| --- | --- | --- | --- | --- | --- | --- |
| Author, year | Population characteristics | Study Design | Exposure Assessment | Outcome Assessment | Covariates | Results |
| In-sook, L., 2008^46^ | n=601  Male: 43.6%  Female: 56.4%  Mean age: 74.2, SD=34.71  Location:  Gyeongnam, South Korea  Setting: Community  Funding: Not reported. | Cross-sectional | The use of assistive devices | Life Satisfaction  Elderly Life Satisfaction Scale | Age  Sex  Income  Marital status  Education  Region of residence  Living arrangements (living alone vs with others)  Subjective health  ADL/IADL  Health related quality of life  Satisfaction with device | No association between the use of assistive devices and life satisfaction ($\hat{\beta}$ = 0.014, p < 0.05, not significant). |
| Leung, V. et al., 2005^47^ | n=5,395  Male: Not reported.  Female: Not reported.  Mean age: Not reported.  Location: Canada  Setting: community  Funding: The National  Health Research and Development Program (NHRDP) of Health Canada, Pfizer Canada Inc., NHRDP,  Bayer Inc.,  The British Columbia  Health Research Foundation,  CIHR | Cross-sectional | Wheelchair use | Life satisfaction  Andrew and Withey Life Satisfaction Scale adapted by Alex Michalos | None | Life satisfaction levels  Among persons with wheelchair was lower than the persons without-wheelchair ($X^{2}$=68.5, p<0.0001). |

Data extraction table for Research Question 2

| Table 2: Characteristics of eligible studies for Question 2, AD and Informal caregiving hours | | | | | | |
| --- | --- | --- | --- | --- | --- | --- |
| Author, year | Population characteristics | Study Design | Exposure Assessment | Outcome Assessment | Covariates | Results |
| Agree et al., 2005^49^ | n = 4,006  Male: 53.4%  Female: 64.6%  Mean age: Not reported.  Location: United States  Setting: Community  Source of Funding: National Institute on Aging | Cross-sectional | Assistive device use | Informal caregiving hours | Number of ADLs with severe difficulty  Insurance  Poverty  Cognitive impairment  Age  Education  Race  Gender  Marital  status  Living environment  Access to healthcare | AD use was significantly associated with reductions in informal care hours, especially for those who were unmarried (AD use $\hat{\beta}$ = 0.14, p < 0.01; Informal care hours $\hat{\beta}$ = -40.17, p < 0.01), better educated (AD use $\hat{\beta}$ = 0.12, p < 0.05; Informal care hours $\hat{\beta}$ = -15.36, p < 0.01) or had better cognitive abilities (AD use $\hat{\beta}$ = -0.17, p < 0.01; Informal care hours $\hat{\beta}$ = 83.77, p < 0.01). |
| Hoenig et al., 2003^51^ | N= 2,638  Male: Not reported.  Female: Not reported.  Mean age: Not reported.  Location:  United States  Setting: Community Funding: American Federation for Aging Research; National Institutes of Health, National Institute on Aging, Duke University, Claude D. Pepper Older Americans Independence Center | Cross-sectional | The use of assistive devices | Informal caregiving hours | ADL impairment  Insurance  Income  Cognitive impairment  Chronic conditions  Age  Education  Race  Gender  Missing hours of help  Hospitalizations | Those who used any technological assistance, either for some or for all of basic ADL impairments, reported 3.8 fewer hours of help per week ($\hat{\beta}$ = - 3.8, p = 0.008) than did those who used no technological assistance. |

## APPENDIX E

AXIS Scoring

In this scoring directly adapted from [Moskalewicz](https://www-sciencedirect-com.proxy.lib.uwaterloo.ca/science/article/pii/S0895435619306912?via%3Dihub#!) et al., the maximum total score achievable is 19 instead of 20.^43^ The minimal score of zero, on the other hand, remains unchanged.

AXIS Scoring adapted from Moskalewicz et al.^43^

| **Questions 1 – 9, 10-12, 15-19, 20** | Yes = 1 point each  No or Don’t Know = 0 points each |
| --- | --- |
| **Question 9** | Yes or not applicable = 1  No = 0 |
| **Question 13 and 14** | If 13 is No, then 14 is considered not applicable/not scored = 1 point  If 13 is Yes, and 14 is Yes = 1 point  If 13 is Yes, and 14 is No = 0 point  If 13 is Don’t Know then 14 is deemed not applicable/not scored = 0 point |
| **Question 19** | No = 1 point each  Yes or Don’t Know = 0 points each |

## APPENDIX F

AXIS Assessment

|  | # | Questions | Research Question 1 Studies (LS) | | Research Question 2 Studies (Hrs) | | |
| --- | --- | --- | --- | --- | --- | --- | --- |
|  |  |  | Leung et al. | In-sook et al. | Agree et al. | Hoenig et al. | |
| Introduction | 1 | Were the aims/objectives of the study clear? | Y - 1 | Y - 1 | Y - 1 | Y - 1 | |
| Methods | 2 | Was the study design appropriate for the stated aim(s)? | PA – 0.5 | Y - 1 | Y - 1 | Y - 1 | |
|  | 3 | Was the sample size justified? | Y - 1 | N - 0 | Y - 1 | Y - 1 | |
|  | 4 | Was the target/reference population clearly defined? (Is it clear who the research was about?) | Y - 1 | Y - 1 | Y - 1 | Y - 1 | |
|  | 5 | Was the sample frame taken from an appropriate population base so that it closely represented the target/reference population under investigation? | Y - 1 | Y - 1 | Y - 1 | Y - 1 | |
|  | 6 | Was the selection process likely to select subjects/participants that were representative of the target/reference population under investigation? | Y - 1 | Y - 1 | Y - 1 | Y - 1 | |
|  | 7 | Were measures undertaken to address and categorise non-responders? | Y – 1 | N - 0 | Y – 1 | Y - 1 | |
|  | 8 | Were the risk factor and outcome variables measured appropriate to the aims of the study? | Y - 1 | Y - 1 | Y - 1 | Y - 1 | |
|  | 9 | Were the risk factor and outcome variables measured correctly using instruments/measurements that had been trialled, piloted or published previously? | Y - 1 | Y - 1 | N/A - 1 | N/A - 1 | |
|  | 10 | Is it clear what was used to determined statistical significance and/or precision estimates? (e.g., p-values, confidence intervals) | Y - 1 | Y - 1 | Y - 1 | Y - 1 | |
|  | 11 | Were the methods (including statistical methods) sufficiently described to enable them to be repeated? | Y - 1 | Y - 1 | Y - 1 | Y - 1 | |
| Results | 12 | Were the basic data adequately described? | Y - 1 | Y - 1 | Y - 1 | Y - 1 | |
|  | 13 | Does the response rate raise concerns about non-response bias? | N – 1* | DK - 0 | N – 1* | Y - 1 | |
|  | 14 | If appropriate, was information about non-responders described? | N | N | N | Y | |
|  | 15 | Were the results internally consistent? | Y - 1 | Y - 1 | Y - 1 | Y - 1 | |
|  | 16 | Were the results presented for all the analyses described in the methods? | Y - 1 | Y - 1 | Y - 1 | Y - 1 | |
| Discussion | 17 | Were the authors' discussions and conclusions justified by the results? | N – 0 | Y – 1 | Y – 1 | Y – 1 | |
|  | 18 | Were the limitations of the study discussed? | PY – 0.5 | Y - 1 | Y -1 | Y - 1 | |
| Other | 19 | Were there any funding sources or conflicts of interest that may affect the authors’ interpretation of the results? | N – 1* | DK - 0 | N – 1* | DK - 0 | |
|  | 20 | Was ethical approval or consent of participants attained? | DK - 0 | DK - 0 | DK - 0 | Y - 1 | |
| Total | |  | 16 | 14 | 18 |  | 18 |
| Mean appraisal score | |  | Research Question 1 | 16 | Research Question 2 | 18 | |
| Key: Y="Yes", N="No", DK="Don't know" | | | | | | | |
| *Item is reverse scored (i.e., no is a positive) | | | | | | | |
